# Young people’s perception of sexual and reproductive health services in Kenya

**DOI:** 10.1186/1472-6963-14-172

**Published:** 2014-04-15

**Authors:** Pamela M Godia, Joyce M Olenja, Jan J Hofman, Nynke van den Broek

**Affiliations:** 1 Division of Reproductive Health, Ministry of Health, P.O. Box 30016, Nairobi, Kenya; 2 School of Public Health, University of Nairobi, P.O. Box 19676, Nairobi, Kenya; 3 Liverpool School of Tropical Medicine, Pembroke Place, Liverpool L3 5QA, UK

## Abstract

**Background:**

Addressing the Sexual and Reproductive Health (SRH) needs of young people remains a big challenge. This study explored experiences and perceptions of young people in Kenya aged 10–24 with regard to their SRH needs and whether these are met by the available healthcare services.

**Methods:**

18 focus group discussions and 39 in-depth interviews were conducted at health care facilities and youth centres across selected urban and rural settings in Kenya. All interviews were tape recorded and transcribed. Data was analysed using the thematic framework approach.

**Results:**

Young people’s perceptions are not uniform and show variation between boys and girls as well as for type of service delivery. Girls seeking antenatal care and family planning services at health facilities characterise the available services as good and staff as helpful. However, boys perceive services at health facilities as designed for women and children, and therefore feel uncomfortable seeking services. At youth centres, young people value the non-health benefits including availability of recreational facilities, prevention of idleness, building of confidence, improving interpersonal communication skills, vocational training and facilitation of career progression.

**Conclusion:**

Providing young people with SRH information and services through the existing healthcare system, presents an opportunity that should be further optimised. Providing recreational activities via youth centres is reported by young people themselves to not lead to increased uptake of SRH healthcare services. There is need for more research to evaluate how perceived non-health benefits young people do gain from youth centres could lead to improved SRH of young people.

## Background

Adolescents (10–19 years) and young people (10–24 years) constitute 18% and 26% of the world population respectively [1]. Investing in the health of young people is essential for the economic and social development of any nation [2]. Young people from sub-Saharan Africa are more at risk of experiencing sexual and reproductive health (SRH) problems than other youth from around the world [3]. The highest adolescent childbearing rates are seen in Africa [4] where young people also have the highest unmet need for contraception [5]. Youth from sub-Saharan Africa face the greatest risk of sexually transmitted infections (STI). Over half of all new HIV infections occur among young people, with girls being four times more likely to be infected than boys [3]. In spite of this, condom use is still very low and uptake of testing for HIV is only slowly increasing [6]. In Kenya, the majority of young people (90%) know where to obtain an HIV test but less than half have ever done so [7].

The World Health Organisation (WHO) recognises the importance of the public health sector in improving the health of adolescents, although the service provision process is often *ad hoc,* and recommends the use of the “4-S framework” in improving the way in which governments address the health problems of adolescents. The framework entails i) gathering and using strategic information, ii) developing supportive, evidence-informed policies, iii) scaling up the provision and utilization of health services and commodities, and lastly iv) strengthening action and linkages with other government sectors [8].

### SRH service provision of young people in Sub-Saharan Africa

The provision of SRH services to young people across sub-Saharan Africa is commonly via public or Ministry of Health, non-governmental (NGO) or faith-based organisations. Two approaches are commonly used for the delivery of SRH services to young people: 1) the targeted (youth-only) and 2) the integrated approach [9,10]. The targeted approach is where services are designed and planned specifically for the use of youth alone and these can either be facility-based, school-based or community-based. In the integrated approach, young people receive SRH services together with the general public in health care facilities but special arrangements are put in place to make the services more acceptable to young people. This may include training of health care providers and improvement of the infrastructure of the health facility or extension of opening times.

There is lack of scientifically sound data on the effectiveness of services targeting young people in sub-Saharan Africa, in comparison to the magnitude of the SRH problems experienced in the region [11,12]. The majority of rigorously evaluated interventions have focused on HIV/AIDS prevention and control [13]. Systematic reviews suggest that current interventions tend to have significant positive effects on improving young peoples’ knowledge and sometimes attitudes regarding sexual behaviour but are less effective in demonstrating change in sexual behaviour outcomes [14-16]. There are suggestions in the literature that interventions fail to produce results because they lack understanding of and sensitivity with regard to the existing community norms and beliefs [17,18]. Most studies have had methodological deficiencies and very few have been able to measure the effects of inventions using biological outcomes such as HIV, STIs and pregnancy rates. For example, a systematic review of HIV interventions conducted in South Africa to identify which interventions worked produced inconclusive results. A definite assessment of *“what works”* was not possible due to limitations in the effectiveness of most interventions and weakness of the study design [17]. Interventions having multiple components such as a combination of health service provider training, facility improvement initiatives and community wide health education activities are often reported to lead to increased service use, although there is a need for more systematic monitoring and evaluation through operations research [19].

### SRH Service provision for young people in Kenya

The provision of SRH services to young people in Kenya is mainly done via three types of service providers: Public or Ministry of Health Managed Services, Non-Governmental Organisations and Faith Based Organisations (FBO). There is an explicit policy framework for the implementation of adolescent SRH services. The National Reproductive Health Policy and Strategy, [20,21] and the Adolescent Reproductive Health and Development Policy and Plan of Action [22,23] both identify adolescent and youth SRH as a key priority component and outlines key priority actions to be instituted to address the SRH problems of adolescents. The national guidelines broadly identify two approaches to be used in the delivery of SRH services to young people: the targeted (youth-only) and the integrated approaches (youth seen within the wider health service system) [9]. Availability of youth-friendly services (YFS) is assessed at national level using three main indicators: i) proportion of facilities with at least one health service provider (HSP) trained in YFS, ii) proportion of facilities with observed policy/guidelines on YFS and iii) proportion of facilities offering youth-friendly HIV testing services [24]. Current estimates show that only 7% of facilities are able to provide youth friendly HIV counselling and testing services, a decline from the 12% of facilities reported in 2004 [25]. Confidentiality, short waiting time, ability to obtain all services at one site and HSP attitude are rated important YFS characteristics by young people in Kenya, although girls are more likely than boys to rate a particular characteristic as “very important”.

This study was designed to explore the SRH problems young people face as well as their perceptions of available SRH services in Kenya. We compared experience of use of integrated and youth targeted SRH services.

## Methods

### Selection of study sites

This was a qualitative study whose purpose was to gain a deeper understanding of the SRH problems young people face and document perceptions of available SRH services as reported by young people themselves. The study took place in four regions: the capital city of Nairobi and three districts (Laikipia, Meru Central and Kirinyaga). A total of nine facilities were purposefully selected with the aim of including interviews with youth from facilities offering youth-only services (3) as well as from facilities offering integrated SRH services (6). Facilities selected in Nairobi included five health facilities offering integrated services and one community-based youth centre. At the district level, the sample included two district hospitals which had facility-based youth centres (Laikipia and Meru) and one district hospital where SRH services for young people were integrated with regular health service provision.

### Selection of young people

Purposive sampling was used in the selection of young people (male or female, aged 10–24 years) seeking SRH services at a health facility and/or youth centre. In addition, young people were selected from the community within the catchment area of the selected facilities by using leaders of existing youth social networks such as youth groups and church leaders. This was particularly important for boys as the number of boys seeking care at the health facilities was low. Leaders of youth groups linked to health facilities helped identify young people who then identified other young people between the ages 10–24 (snowball technique).

### Data collection methods

Focus group discussions (FGD) and semi-structured in-depth interviews (IDI) were used as methods of data collection. The research tools were translated to Kiswahili, the national language of Kenya. FGDs and IDIs took place at both health facilities and in the community. At health facilities, FGDs took place in a room that had been allocated to the research team. In the community, the interviews took place at a designated community church or hall. The FGDs and IDIs were conducted in Kiswahili language, although respondents tended to mix Kiswahili and English languages during the interviews. All FGDs were conducted with two researchers; a moderator and a note taker. The moderator was responsible for guiding the discussion while the note taker was responsible for taking notes, noting down non-verbal responses and ensuring that tape-recoding was on-going. Each FGD took between 90–120 minutes and consisted of between 6–10 participants. FGDs were held separately for girls and boys to allow for free expression of views during the discussion of potentially sensitive issues [26]. IDIs were conducted by a single researcher and took between 30–45 minutes. The researcher also made sure the IDIs were tape-recorded and took short notes as the interview progressed. Both FGDs and IDIs explored the following topics: SRH problems young people face, health seeking behaviour, views and perceptions of available SRH services, barriers to and facilitators of seeking health care and suggestions for improving access and utilisation of services.

### Data management and analysis

Transcription of the data collected in this study was done by the research assistants under the guidance of the principal researcher. The verbatim transcription was done directly into English from Kiswahili. Initially the plan was to have the tape-recorded interviews first transcribed in Kiswahili and then translated into English but it became apparent that the process would take longer and the cost would be beyond our means. A decision was then made to have the tapes carefully transcribed directly into English. This was also supported by the fact that members of the research team were conversant with both English and Kiswahili languages. Moreover in most instances, respondents mixed the two languages during the interviews. The transcripts were typed out and each transcript saved as an individual word document with clear labelling showing the study site, type of interview and respondents sex. Transcript for IDIs of young people aged 10–14 were analysed separately to bring out their views and experiences

Once the transcripts were translated to English, they were saved on a password protected computer only accessible to the research team. An independent researcher conversant in both Kiswahili and English, cross-checked the transcripts for accuracy and language translation consistency [27]. Trustworthiness of the data was met through triangulation of three aspects of data collection: i) having different respondents, ii) using different methods of data collection such as IDIs and FGDs, iii) using different researchers with different experiences to conduct interviews and moderate group discussions [28]. The data was validated through validation meetings with some of the respondents who took part in the study. NVIVO8 was used to guide the data management and coding of the transcripts. Data analysis was conducted using the thematic framework approach.

### Ethical considerations

In the IDIs, informed consent was obtained from each young person, who signed a consent form, after a detailed explanation about the purpose of the study had been given. With regards to FGDs, verbal consent was obtained from all group members and only one consent form signed by the FGD moderator to signify the group’s acceptance to participate in the study. This was done after an explanation had been given to the group members about the purpose of the study and the importance of their views as service users. Respondents were also given information sheets which had details about the purpose of the study. Respondents were assured of privacy and confidentially and that the data collected would only be accessible to the research team. Participants were informed of their right to refuse to participate in the study or withdraw from the discussion at any time. For young people 15 years and below, verbal consent was obtained from either the parents or guardians but the adolescents thereafter confirmed their agreement to take part in the study by signing a consent form.

Ethical clearance and approval was obtained from the Liverpool School of Tropical Medicine Research Ethics Committee in the United Kingdom and the Kenyatta National Hospital Ethics and Research Committee in Kenya.

## Results

A total of 18 FGDs, were held with young people aged between 15–24 years, 10 with girls and 8 with boys. With regards to facility type, 13 FGDs were held with young people from integrated facilities and five with young people from youth centres. A total of 39 IDIs were conducted with young people out of whom 15 were boys and 24 girls, aged 15–24 yrs and 8 were aged 10–14 yrs (8). Twenty three young people were from integrated facilities while 16 were from youth centres (Table 1). Only IDIs were held with younger adolescents (10–14) as their numbers were not adequate to constitute an FGD. More young people were interviewed from integrated facilities than youth centres because it is the most common service delivery model available. There are other youth centres outside Nairobi and in other districts but these were not visited due to logistical and financial limitations.

**Table 1:** Details of young people who participated in FGDs and IDI

**Young people who participated in FGDs (18)**				
**Study site**	**Sex of the respondents**		**Service type**	
	**Girls**	**Boys**	**Integrated**	**Youth centre**
Nairobi	8	5	11	2
Districts	2	3	2	3
**Total**	**10**	**8**	**13**	**5**
**Young people who participated in IDIs (39)**				
**Study site**	**Sex of the respondents**		**Service type**	
	**Girls**	**Boys**	**Integrated**	**Youth centre**
Nairobi	16	8	20	4
Districts	8	7	3	12
**Total**	**24**	**15**	**23**	**16**

The study findings are reported according to the themes that emerged from the data:

a) SRH problems faced by young people

b) Addressing the SRH needs of young people

c) Perceptions of existing SRH services

d) Suggestions on how to improve SRH services

## SRH problems faced by young people

**Figure 1:**
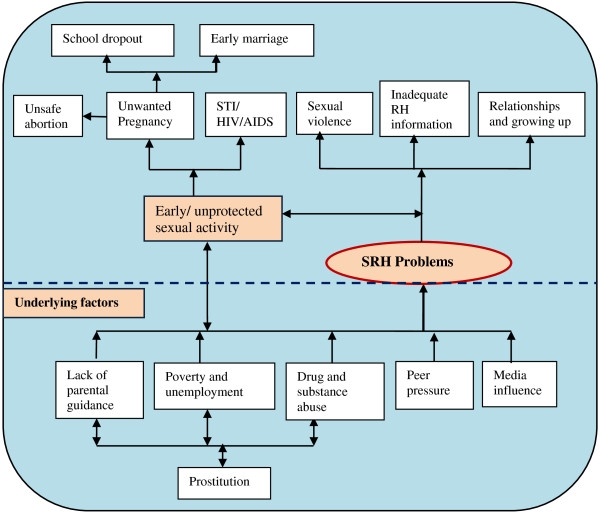
Young people’s perceptions of SRH problems and needs.

Although young people were initially asked to identify which common SRH problems they experienced, their responses were broad and reflect the cultural, social and economic environment in which they live (Figure 1). Problems commonly mentioned included: early and unprotected sex, unwanted pregnancy, infection with STIs including HIV/AIDS, unsafe abortion, sexual violence and female genital mutilation. Other concerns included inadequate information on reproductive health generally, problems related to physical body change during the period of adolescence and relationship problems. Social problems which young people considered important, included lack of parental guidance on sexuality and growing up, poverty and unemployment, drug and substance abuse, media influence and peer pressure. Problems mentioned infrequently included prostitution, early marriage, and school drop-out.

Across the study sites, the SRH problems mentioned by girls and boys from both Nairobi and the districts were similar with no major variations on their views on why they underwent such experiences. Poverty and unemployment were said to heavily influence whether young people engaged in risky reproductive health practices. Young people implied that *“health issues”* that affected them came about *“due to poverty”* coupled with unemployment which was perceived to lead to feelings of hopelessness.

For example, financial and material gains were identified by both girls and boys in all the study sites as major reasons why young people, particularly girls, got involved in early and unsafe sex practices, including having multiple and older sexual partners. This was particularly said to happen among girls who were orphaned, poor and had to find a source of income. This was commonly referred to as *“sex-for-maize-flour”*. Some girls eventually ended up engaging in prostitution as the exchange of money for sex became a part of their daily routine.

*“Poverty, let’s say the girl comes from a very poor family, so she will want to look for money so that she can help her family, and if she gets a boy* [boyfriend] *who has a bit of money, she will be forced to go and do what is supposed to be done* [have sex]*, without preventing anything* [using any protection]; s*o that at least she can get the money and take it home, so that her siblings can get a little food”,* (FGD-boys, Nairobi).

Media influence on young people’s sexual practices, especially pornographic material, was mentioned by majority of boys from all the study sites. However, pornography was rarely discussed among girls as they said they did not often browse the internet. Most television programmes were said to have some pornographic content which perpetuated sexual activity among young people; pornographic material was said to be easily available on the internet particularly through the mobile phones.

*“…that habit* [sex] *is also enhanced by these movies, because 80 percent of TVs* [programmes] *have pornographic movies in them…....but also even the mobile phone; this is the thing where the young people are actually accessing everything.....”* (FGD Boys, Meru district)

Unplanned pregnancy among adolescent girls was a SRH problem that was mentioned across all the study sites by majority of girls and boys, with no variation in views between young people from Nairobi and the districts. Girls were said to experience unwanted pregnancy due to financial reasons and if they had disagreements with their parents.

In the FGDs with girls from Nairobi, there were concerns about boys *“denying”* or *“disowning”* girls they made pregnant by refusing to take up responsibility. This was reaffirmed by boys who stated that denial of responsibility happened if the pregnancy occurred *“accidentally”, “unplanned”, “unexpectedly”* or by *“bad luck”* and the boy had no means of supporting both the child and the girl financially. This was said to be a major burden to girls as they were left to fend for the child on their own. Boys also said that they were pressurized and forced by their families to deny such responsibilities. This was summarized by one respondent who said that:

*“What is there is that boys are so willing to have sex, but they are not responsible to take the outcome afterwards, about sex they are ready, even yesterday, but if they are told; yes you have had sex and this are the results can you take the responsibility? Most boys are not in that situation. The boys will not take it, they will not be ready”,* (FGD boys, Nairobi)*.*

Similar views were expressed by girls in Laikipia district.

*“…once a girl gets pregnant and the boy maybe they had not planned, the boy will deny, then the girl will get a problem of taking care of the child alone”,* (IDI girls, Laikipia).

Both girls and boys from all study sites reported lack of keenness, among young people, in knowing their partner’s HIV status before engaging in unprotected sex. In the in-depth interviews, a boy in Nairobi, expressed concern that some of the new cases of HIV were occurring among young people who had received information about HIV/AIDS. The reasons given for engaging in risky sexual activities were flimsy such as *“niliteleza”* meaning “*I tripped”* (by mistake, bad luck).

In the FGDs, young people expressed concerns of a trend among youths whereby, when involved in a new sexual relationship, they would use the condom during the initial three or four sexual encounters. Thereafter the issue of trust sets in and young people start having sex without protection before having medical tests like HIV testing and counselling.

*“There is this tradition that if you use a condom for 3 to 4 times, trust comes in. You end up saying okay we trust each other now, so we can have sex without a condom. So you will find that most young people, once they use the condom maybe three or four times, the fifth time they will have sex they will not use a condom”, (*FGD Boys, Nairobi)

*“…because like the youths do not consider going to the VCT for test so when somebody comes to approach you, you just assume they are alright so you end up messing yourself because he will have sex with you and transmit the diseases to you”,* (IDI girls, Kirinyaga).

The discussions from both girls and boys in all the study sites indicated lack of proper parental guidance as being the genesis of some of the SRH problems young people experienced. Parents were said not to listen, talk to and advice their children appropriately on issues such as menstruation (when it begins and personal hygiene), consequences of unprotected sex because, either they did not have time, or were afraid and uncomfortable having such discussions or due to cultural limitations. Lack of proper understanding or open communication channels between parents and their children was highly emphasized by boys and girls from all study sites.

*“There is lack of contact between the parents and the children, especially when it’s about sex, you see, us young people learn those STDs from our peers, we have lost contact with our parents----”* (FGD Boys, Kirinyaga district).

*“Another challenge for the youth is the time when we get to adolescent age, our parents fail to teach us how to behave, if it is a girl, she is supposed to be told that if you do this and this, there will be repercussions that is why you find that young girls are becoming pregnant at an early age, that is why some are contracting diseases outside there because our parents are failing to teach us and it ends up badly for our children”,* (FGD girls, Nairobi).

### Addressing the SRH needs of young people

Young people were asked to make suggestions on how some of the SRH problems they had identified could be addressed. Both boys and girls mentioned the use of contraception including condoms and traditional methods (safe days) as ways of preventing unwanted pregnancy. With regards to STI and HIV/AIDS prevention, condom use, abstinence and not having many sexual partners were mentioned. Young people generally wanted more accurate SRH information especially from parents.

#### Contraception and family planning

Although the majority of boys and girls across all study sites knew that contraceptives were effective, they believe that girls should not be given hormonal contraceptives (injections and oral contraceptives) if they have not yet had a baby as this would prevent girls from conceiving in future. The majority view was that hormonal contraceptives should be reserved for married women or girls who had given birth to at least one child. The use of the word *“family planning”* was reported (especially by boys) as “off putting” as it implies this is for people who already have or want to start a family and therefore does not include young people especially if they are not in a long-term partnership.

The majority of girls from Nairobi believed that *family planning “medication” “accumulates”* in one’s *“body”* or *“stomach”* and ends up *“spoiling you”,* especially when used over a long time. Among pregnant girls who had come for antenatal care (ANC) none had used hormonal contraception before conception of their first child.

#### Young people and condom use

Boys and girls from all study sites expressed conflicting views about condom use. Young people did not talk about their personal experiences, but referred only to condom use by a third party; *“other youths”, “other people”.* Condom use was said to be good as it protected one from STIs (especially HIV) and early pregnancy. The benefits of condom use were said to be immense especially as young people had many sexual partners and/or short-term sexual relationships. Young people reported that very few of them used condoms consistently. Reasons given for this included lack of societal support, not knowing how to use condoms, refusal by some girls, lack of sexual satisfaction and the belief that condoms were not.

*“They hate using condoms because they say it’s like eating a sweat in its wrapper. They also say a condom is not 100% assurance that one can contract HIV”,* (IDI-18 yrs boy Kirinyaga district)

*“Some say maybe when you use a condom…..some maybe it can burst, or maybe it can have some holes, it can burst in the process of using it”,* (IDI-19 year old girl, Meru district)

*“They say it is not good to use condoms and others they say condoms are for prostitutes, people who have no relationships”,* (IDI-23 year old girl, Meru district, service user)

*“Some say they do not use because it has tinny holes below it, if you sleep with a person you may get a disease”,* (IDI-22 year old girl, Nairobi)

*“There are some who say it is bad, it can burst or remain inside your stomach, it later brings you problems in the tummy later, you stay in hospital”,* (FGD-girls, Nairobi)

Knowledge of younger girls (12–14 years) was limited with majority reporting that they did not know much about condoms. Boys the same age were more knowledgeable and reported that young people used condoms for prevention of HIV, pregnancy and other STIs. A minority of boys reported that although the use of condoms was taught at school, they were still advised to abstain from sex until marriage.

#### Young people and pregnancy prevention responsibility

Most boys in Nairobi suggested that it was the responsibility of the girl to ensure that she prevented herself from getting pregnant and that they found it difficult enquiring or initiating discussions with girls about contraceptive use.

*“You know a boy does not care, when I have finished with her* [had sex] *I just go my way, if she wants to get pregnant or she does not want, it is her problem”,* (FGD Boys, Nairobi).

The majority of boys from all study sites also suggested that some girls actually wanted to get pregnant out of their own desire and at times pregnancy would be used as a tool for securing commitment from their boyfriends, more so, if the boy had a good job and was financially stable. A few boys in Nairobi indicated that some girls chose to become pregnant due to peer pressure. Such girls regarded pregnancy to be a *“fashion”* or *“class thing”* and therefore became pregnant intentionally as a way of identifying with their peers; a statement that was refuted by most the girls during their FGDs.

*“Yes it is like fashion because if they sit around, you hear them saying; “you do not have a baby, what are you waiting for?” So to them, they see as if it is a normal thing, they do not see it as something surprising”,* (FGD-Boys, Nairobi)

*“It is not that girls want to get pregnant, No, you cannot leave your house and say today I am going to get pregnant, can you do that really? And you are still in school?”* (FGD-Girls, Nairobi).

#### Parental guidance and sex education

In all study sites, both boys and girls emphasised the need for parents to give their children “proper guidance and education” on matters related to sexuality, growing up and reproductive health generally. Parents need to talk with openness and give factual information without being evasive. Young people stated that they placed great value on advice given by parents as they were likely to remember such advice in adulthood. Girls reported the need for parental advice on matters concerning menstruation such as proper use of sanitary towels, personal hygiene and how to handle painful menstruation. They also needed advice on prevention of unwanted pregnancy and STI/HIV. Girls expressed the opinion that it was their right to receive such advice from parents especially mothers and they felt that absence of such discussions negatively affected the formation of cordial mother-girl relationships, resulting in difficulty in initiating personal discussions with parents, when the need arose.

*“In our families, parents should open up to their children and start to educate them because this is not like the old days, you should just talk to your kids and … it should start in the family; parents should be open to their kids”,* (FGD girls, Nairobi).

A minority noted that parents were *“too strict and harsh”* and did not give them a chance to make decisions independently or socialise with other young people. It was suggested that parents should not *“enclose”* their children but allow them to interact and socialise with their friends under *“logical restrictions”.* Girls from Nairobi said that parents made assumptions that young people were aware of certain reproductive health information when, in reality they were not. Young people also reported that biology learned in school was not a substitute for getting information and advice about their reproductive health from parents.

### Perception of existing SRH services

Perceptions of services were both positive and negative and differed for integrated health care facility or youth centre (Table 2). In Nairobi girls visiting integrated facilities for ANC or contraceptives reported the services as good, affordable and helpful with positive interaction with Health Service Providers (HSPs). Majority of pregnant girls from Nairobi stated that HSPs were cordial, friendly, welcoming and helpful. They provided good advice on pregnancy care and nutrition.

**Table 2:** Young people's views and experiences of available Sexual and Reproductive Health services

**Facility type**	**Gender**	**Positive views and experiences of SRH services**	**Negative views and experiences of SRH services**
**Integrated facility**	Girls	▪ Good and friendly services	▪ HSP attitude sometimes judgmental
		▪ Positive health service providers’(HSP) attitude	▪ Provider gender – not always female
			▪ Long waiting time – long queues
		▪ Low cost of services	▪ Lack of essential drugs
			▪ Corruption in health facilities
	Boys	▪ Organisation of services – services perfect for women and children	▪ HSP attitude poor
			▪ Lack of awareness of available services
		▪ HSP attitude is positive	▪ Youth-related resources not available such as library, games
		▪ Cost of services – affordable, waiver system available	
			▪ Provider’s gender not always male
		▪ Facility is clean	▪ Long waiting time
		▪ Qualified HSP	▪ Corruption in public health facilities:
		▪ Facility location- walking distance	▪ Lack of proper directions to facility and service areas
			▪ Lack of privacy
			▪ Boys feel uncomfortable sitting between women
**Youth centre (YC)**	Girls	▪ Good services - youth-only	▪ Lack of full-time clinical staff
		▪ Health service providers are younger	▪ SRH services not provided around the clock
		▪ Non-health benefits of participation in YC activities – personal gains, self development, career progression	▪ Occasional lack of supplies such as drugs
			▪ Lack of awareness of services offered
		▪ Youth centre attractiveness promotes step-wise use of services	
	Boys	▪ Wide range of services available: voluntary counselling and testing (VCT), treatment of STIs, contraception, counselling, games, educational films watching movies	▪ Limited hours of operation for public facilities
			▪ Inaccessibility – with regard to public transportation (YC location)
			▪ Lack of essential supplies
		▪ Health provider attitude – positive	▪ Lack of awareness of available services
		▪ Youth centre attractiveness	
		▪ Non-health benefits of the YC	▪ Poor system of getting updated SRH information

Boys said that services available at the integrated health centres were serving the needs of women and children well.

*“If we talk about services here* [integrated health centres in Nairobi], *mostly we will not talk about youths, we will mostly talk about mothers.....we can say the services here are smart.......on the side of mothers the services are perfect”* (FGD boys, Nairobi).

The majority of boys from integrated facilities reported an improvement in most public health facilities such as facility renovation, better HSP attitude/approach and relationship with clients. Public health facilities were perceived to have more qualified staff compared to some private health facilities in urban slum areas.

*“What I can say about the services of this* [integrated health centre] *for the last 3 years, there has been an improvement; its services have been of good quality. Because earlier on even coming to a public clinic was very difficult. First of all the way the nurse would treat you, you would not like even to go in there, you are sick and here you are being harassed but nowadays they have really improved”* (FGD Boys, Nairobi)

Similar sentiments were expressed by boys from the districts,

*“Nowadays I can say at least the government has done a lot, at least it has facilitated some trainings maybe to teach* [health providers] ----- *the* [district] *hospital has changed a lot, even the way service providers are talking to and handling clients”* (FGD Boys, Meru district).

Boys and girls outlined both health and non-health benefits of youth centres although the majority reported that the benefits of using a youth centre were mainly non-health related. Youth centres were described as *“good”, “friendly”, “open”, “useful” “helpful”* with HSP who were young, friendly, understanding and easier to talk to.

*“I think it is a good place for us to go because it is not like the other place* [−general health facility -]…*where most people go but this one* [−youth centre-] *is for the youths only and some of the staff working there are youths, like us, and they understand what we go through, so it is easier working with them”,* (IDI 14 year old girl, Meru)

Youth centres provided a good environment where privacy was respected and there was friendliness among staff. HSP gave advice to young people on how to plan for their future lives and prevent unwanted pregnancy and early marriage. The counselling services were good especially in situations where parents were unable to inform their children about RH matters.

*“They provide everything, I can go there to play, to watch movies, I can be guided, I can be tested*”, (IDI 14 year old, Laikipia youth centre)

The other reported benefits included engaging in recreational activities and provision of information, vocational training and advice on career progression. The consensus opinion was that young people who came to the youth centre to play games or be involved in other activities eventually would end up using the centre’s SRH services if needed. Activities at the youth centres were linked to building young people’s confidence in terms of improving self-esteem, communication skills and general interpersonal interaction with peers and members of society as well as allowing them to evaluate their moral values. Girls and boys appreciated the opportunity to receive computer training, learn how to access the internet and write CVs.

*“To me, what I can say is the youth centre has really helped me because when I left school I could barely talk in front of people but you see, the things I have encountered in this youth center, I have at least learned to stand up and talk in front of people. I have known what I did not know when I come from school, as in how to speak myself how to take care of my own body, what I can do so that I can keep away from negative things I always stay positive. How I can face the challenges in life, the centre has really helped me”* (IDI-19 year old girl, Nairobi)

*“Most of them appreciate it* [youth centre] *because it builds their moral values, some of them, others they use it as a stepping stone so that they can have a brighter future. So most of them say it is a good facility in our area where the youth can access services, can use it as a stepping stone to access other information….”* (IDI-19 year old boy, Nairobi)

### Reasons why young people do not seek SRH services

**Figure 2:**
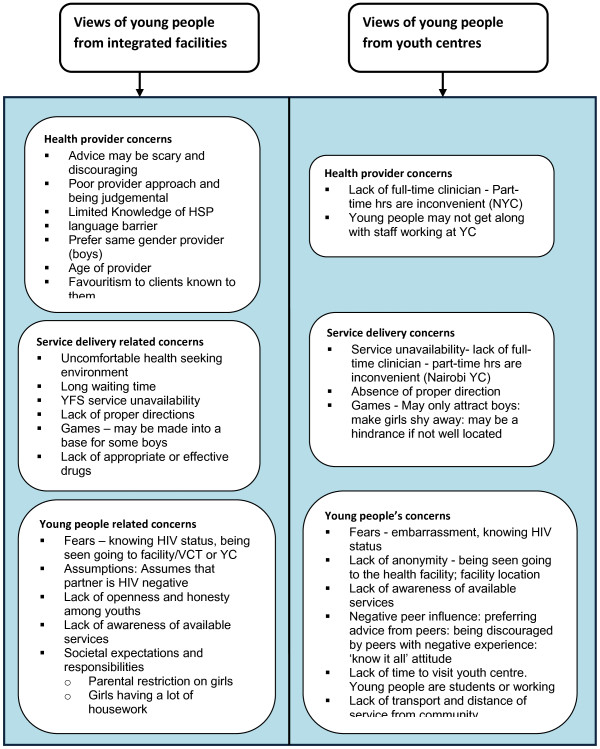
Young people's reasons for not seeking health services.

The majority of boys and girls from all study sites indicated that HSP attitude had great influence on their uptake of and satisfaction with SRH services. Girls described how “simple” things really mattered to them such as: HSP reception, facial expressions, simple greetings, being given the chance to express themselves and explain their problems. The majority of boys and girls were concerned about long queues that were present at some healthcare facilities. Boys reported being impatient and wanting “quick attention” without being lectured at or “tossed” from one hospital department to another (Table 2 and Figure 2).

The majority of boys indicated that the layout at integrated health care facilities, including the waiting area, has been designed for women and children. Boys stated that they did not feel comfortable sitting in the waiting area, *“between women”.* The boys indicated that services at the integrated facilities were perfect for women and girls; but boys had been neglected by the system.

Young people’s expressed two main concerns with regard to youth centres; lack of full-time clinical staff and they questioned the added value of having games at youth centres. Young people were concerned that lack of a full-time clinician resulted in missed opportunities.

*“I think most of the time the nurse is not available, she comes thrice in a week, so if you get a problem on the day she is not here, this is a problem”* (FGD Girls, Nairobi youth centre).

There were conflicting views about the importance of games. While the consensus was that games prevented idleness, it was also felt that games (such as pool) could turn a youth centre into a meeting base for particular groups of boys. Both girls and boys noted that games such as pool only attracted boys and made girls shy away from coming to a youth centre. Also youth playing games at the same place where health services are provided can be a deterrent:

*“So if you come … you will find your friends. If you decide to go and see a healthcare provider,* [these friends] *will be waiting to ask you what had you gone to do? So that thing* [pool table] *needs to be put right* [at the back] *and these other services in front”* (FGD Boys, Nairobi).

### Suggestions for improving SRH services

Young people had a range of suggestions (Table 3) regarding SRH service availability. The majority wished to see an increase in SRH services especially in rural areas including the use of mobile clinics. The consensus was that providing a wide range of SRH services in either integrated health facilities or youth centres was more likely to ensure anonymously and that privacy could be maintained. Girls also mentioned having *girl-talks, girls-days and “things to do with beauty”* as a way of attracting girls.

**Table 3:** Suggestions from young people on how to improve sexual and reproductive health (SRH) services

**Gender**	**Youth centres**	**Health centres/or integrated facilities**
**Girls**	**Increase of SRH service availability**	**Increase of SRH service availability**
	▪ Full-time clinician	▪ More services to be made available at facility (lab), maternity
	▪ Available medication (STI)	▪ Facility to be neat, attractive and well organised
	▪ Establish more youth centres, especially in rural areas	**Improve HSP attitude**
	**Improve HSP attitude**	▪ Improve HSP approach & attitude:
	▪ HSP to be more friendly	▪ HSP to be more patient and friendly, not very elderly
	**Create awareness**	**Create awareness**
	▪ Sensitize YP on importance of youth centre through fun-activities, radio, posters, music entertainment, churches,	▪ Advertise through radio
		▪ Tell peers about service availability
	▪ Encourage openness among adolescents	▪ Awareness creation on importance of services and VCT through seminars in churches, schools, community
	▪ Advertise services through outreaches in community, *have girls-talks*	
	▪ Give out educational materials, booklets, magazines	▪ Organise community meetings: inform parents what goes on at youth centre
	▪ Other resources - have library, games	Have a suggestions box
	*Note: Girls not able to give suggestions (12 yrs and 14 yrs)*	Reduce waiting time
		*No response from some girls in one HC in Nairobi (FGD 05c on how services can be improved*
**Boys**	**Increase SRH service availability**	**Increase SRH service availability**
	▪ Set-up more youth centres	▪ Wide range of services in same room
	▪ MOH to be involved in addressing SRH of young people, support youth activities, peer education	▪ Have proper service directions
		▪ Have youth-only rooms
	▪ Set-up more services in rural areas (mobile clinics)	**Improve HSP attitude**
	▪ Increase operating hours/night services (VCT)	▪ HSP to be open and helpful
	**Improve HSP attitude**	▪ Confidentiality is important
	▪ HSP to be friendly, kind, patient, polite, confidential, not have very elderly staff	**Increase awareness creation of SRH services**
	**Increase awareness creation of SRH services**	▪ Create awareness of services available, having seminars, free medical campaigns, use of artists
	▪ Create awareness of youth centre – through outreaches, advertising in sporting activities, websites	
		▪ Involve the youth in mobilising other youths/Peer education
	▪ Have youth talk to other youths about the services available	**Reduce waiting time**
	▪ Take the education to the schools	**Have educational materials**
	**Youth centre accessibility**	▪ Have educational materials on SRH problems and treatment options
	▪ Location of youth centre should be accessible, close to public transport	Other resources – have library
	**Have educational materials**	*In one FGD boys were not sure of what can be done to increase access*
	▪ Update educational materials	

*There was a suggestion for the is a need to increase awareness of available SRH services* among young people and the community in general. This could be done through outreach activities in the community, schools and churches. Use of local radio stations, posters, magazines, sporting activities and entertainment were mentioned.

*“outreaches is what will help them* [young people] *because most of them do not know about what* [service] *is at the youth centre……..the youth do not know what kind of youth friendly* [services] *are available”* (FGD Boys, Meru)

*Having up to date educational materials* at health facilities, libraries and other social places which young people frequent was also seen as helpful.

*Extending opening hours* to include weekends, public holidays and evenings were popular suggestions.

*“…we don’t open the youth centre on Saturdays and you find most of the youths in the community are free on Saturdays, so they depend on the centre but in a way they are blocked from using it……so if it is possible to open throughout from Monday to Monday more youths will access the facility and also get more information”,* (IDI-24 year old boy, Meru)

## Discussion

To the best of our knowledge, this is the first paper describing the perceptions of youth themselves regarding the sexual and reproductive health (SRH) services offered in both urban and rural settings in Kenya. Young people’s perceptions of available SRH services are not uniform and show wide variation between boys and girls, as well as, with regard to the model of service delivery. In this study, young girls attending antenatal care and family planning clinics in healthcare facilities providing integrated care reported that these services meet their needs very well. Similar findings have been reported in Mozambique where an evaluation of youth-friendly health services (YFHS) located within public health facilities reported that young women found the services offered met their needs with regards to contraception and ANC [29].

Results of national representative surveys conducted among 12–19 year olds in four African countries (Burkina Faso, Ghana, Malawi and Uganda) showed that the majority of adolescents expressed positive views about services provided within the public health system especially with regards to maintenance of confidentiality, being treated with respect and accessibility and availability of services via the existing health care system [30]. Public health facilities were reported to be the most preferred source of contraceptives and HIV testing among young people from these four countries [31]. An evaluation of adolescent friendly health services (AFHS) in Mongolia among 10–19 year olds, showed that more females than males (76 percent vs. 66 percent) were satisfied with the services [32]. These findings suggest that there is an opportunity to provide acceptable high quality SRH services to adolescents via the existing public health system that should be exploited and that making services adolescent friendly increases utilisation. This could be done by increasing availability of services and improving quality by ensuring for example that the services are adolescent friendly and opening hours are extended.

In contrast to girls, boys perceived the service delivery environment within public health facilities in Kenya to be unfriendly. The apprehensive feelings boys have towards using SRH services in public health facilities could be due to the fact that most services offered within these clinics are indeed more “receptive” to women perhaps because women constitute the majority of clients at these facilities.

In Estonia with a new programmatic approach, the numbers of young people accessing youth centres increased steadily, although the number of males visiting the centres remained low [33]. Innovative ways such as use of telephone and online counselling may need to be explored to reach more young people. The National Adolescent Friendly Clinic Initiative (NAFCI) in South Africa uses improvement of quality methodology such as setting standards and introducing accreditation [34,35]. This has resulted in better quality of services but without a significant increase in service utilization among young people [36].

The findings in this study indicate that although young people describe youth centres as having both health and non-health benefits, they particularly value the non-health benefits including general life skills and skills leading to improved chance of employment received through participation in youth-related activities. The presence of games at health facilities such as a pool table elicits mixed reactions. Games at youth centres seem to favour boys, and may make girls feel unwelcome. Evidence supporting the association between non-health related activities (such as games, other recreational activities) and an increase in uptake of health services is weak [12,30,37]. Systematic reviews show that although a youth centred-approach is popular in developing countries, effectiveness with regards to increasing young people’s use of SRH still remains low [37]. Young people using recreational activities and youth centres are often older [30,37,38]. This concern and limitation was also expressed by the young people interviewed in Kenya in this study.

While young people acknowledged the importance of contraception to prevent unwanted pregnancy, the majority are of the opinion that hormonal contraceptives are detrimental to the health of young girls especially those who have never given birth. Contraception is seen within the context of “child spacing” especially where such terms as “Family Planning Clinic” are used. Other studies have reported similar findings where contraceptive use by young girls was not approved by young people, community members and health service providers because it was considered to affect young girls’ fertility [39,40]. In Brazil young people also reported being concerned about potential side effects of contraceptives on future fecundity [41]. These findings imply that education regarding hormonal contraceptives and messaging or social marketing of these requires renewed attention.

We recognise the limitations of this study which was a qualitative study across several sites in Kenya hence the results presented cannot be generalised to the whole of the Kenyan population or other sub-Sahara African countries. Data on actual service utilisation trends were not collected. Nevertheless, it is likely that there are several shared experiences among adolescents across countries in this region. Focus Group Discussions with young people were not disaggregated by age and marital status categories. There is some evidence that younger adolescents do not have the same perceptions and needs as older adolescents and that marital status affects provision and utilization of SRH services. There is also evidence that health service providers are more receptive to providing contraception to married adolescents who have begun childbearing than those who are single and without any child. This is due to the belief that contraceptives at an early age negatively affect the future fertility of the adolescent. Finally, this study relied mostly on self-reported data which is prone to bias, especially with respect to sexual behaviour.

## Conclusion

This study shows that young people cannot be considered as a homogeneous group and their SRH problems as well as perceptions about available SRH services are diverse and show variation between boys and girls. Public health facilities offering a full range of health care services (including SRH) were generally positively viewed and received approval with regards to SRH service provision for young girls and women. However, boys in particular find this environment uncomfortable because of the way the services are organised including the client flow. Providing SRH services to young people through the public health system (integrated model) presents an opportunity that can be exploited because a large number of public health facilities are in place and geographically spread out nationally.

Evidence linking the presence of recreational services at youth centres and general health facilities to increased access and utilization of ASRH services is insufficient except with regard to non-health related benefits or to act as a “stepping stone” to future career progression. More research on how non-health benefits of youth centres could translate into improved utilization of SRH services by young people is needed.

The Kenyan society is still very conservative with regards to discussion around sex and sexual health because of existing religious, social and cultural norms and values. Future research should involve the design, implementation and evaluation of structural interventions addressing the social, cultural and economic drivers of sexual health of both boys and girls.

## Competing interests

The authors declare no competing interests.

## Authors’ contributions

GMP participated in the study design, data collection and analysis, drafting, reviewing and revising of this manuscript. OMJ participated in the study design, data collection and analysis and revising this manuscript. JJH participated in the study design, data analysis and reviewing of this manuscript. NvdB participated in the design of this study, data analysis and reviewing this manuscript. All authors have read and approved the manuscript.

## Pre-publication history

The pre-publication history for this paper can be accessed here:

http://www.biomedcentral.com/1472-6963/14/172/prepub
